# Management of Vulval Lichen Sclerosus—The Role of the Plastic Surgeon

**Published:** 2019-04-24

**Authors:** Sameer Gujral, James Coelho, Shaheel Chummun, Andrew Watts

**Affiliations:** Department of Plastic Surgery, Royal Devon and Exeter Hospital, Exeter, United Kingdom

**Keywords:** vulval, lichen, sclerosus, skin, graft

## DESCRIPTION

A 45-year-old woman with vulval lichen sclerosus underwent excisional surgery with healing by secondary intent after several treatments of topical steroids failed. Recurrent disease and scarring led to a narrowed introitus, persistent discomfort, and dyspareunia. Plastic surgery referral led to reexcision and split skin grafting (SSG) with improved symptoms and quality of life.

## QUESTIONS

1. What is vulval lichen sclerosus?
2. How does it present and how is it diagnosed?
3. What is the management of vulval lichen sclerosus?
4. What is the potential role of plastic surgery in treatment?

## DISCUSSION

Lichen sclerosus (LS) is a chronic inflammatory dermatosis of unknown cause, resulting in white plaques with epidermal atrophy and scarring. It most commonly affects genital areas (80%-85%) but has extragenital manifestations including wrist creases, breasts, neck, and axillae.^1^ Female to male incidence is 6:1. In women, vulval presentation is common.^1^ Lichen sclerosus is often considered autoimmune, though there may be hormonal, irritant, and genetic components.^2^ Extracellular matrix protein-1 antibodies have been detected in 60% to 80% of vulval LS cases.^3^ Genital LS in men is rare in those circumcised in infancy, suggesting that chronic damage by substances occluded under foreskin may contribute.^4^ Onset in females is commonly prepubertal and postmenopausal, suggesting that a relative estrogen deficiency may play a role. Differential diagnoses include lichen planus, cicatricial pemphigoid, morphea, and vulval intraepithelial neoplasia. There is also a less than 5% risk of malignant transformation of vulval LS to squamous cell carcinoma.^1^

**Figure 1:**
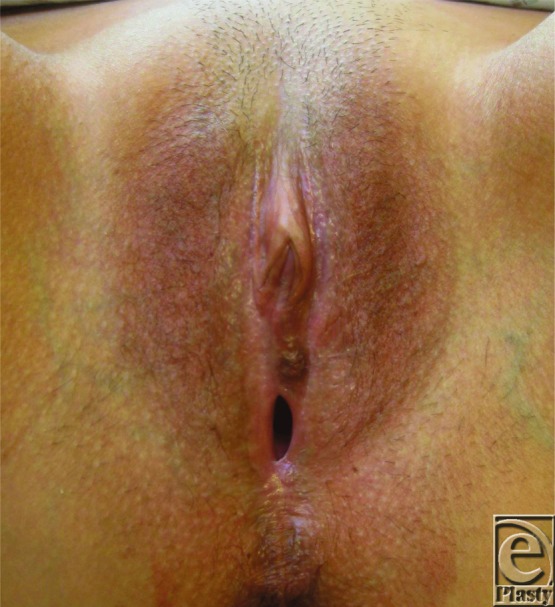
Vulval lichen sclerosus showing white plaques around introitus, labia minora, and clitoral hood. Stenosis of the introitus is visible.

Commonest symptoms are itching and pain. Itching results in fragile skin cracking, bleeding, and then scarring. Presentation in women includes pale plaques around the vagina, vulva, and anus associated with dyspareunia, tightness, altered urinary stream, and constipation. Lichen sclerosus primarily involves non–hair-bearing, inner areas of the vulva. Scarring can be localized or extensively involve perineum, labia minora and majora, clitoral hood, inguinal fold, and anal and perianal skin resulting in labial fusion and clitoral adhesion/burying. Quality of life can be greatly impaired, and patients suffer psychologically and physically. Diagnosis of vulval LS is made from the history, typical appearance of lesions, and histology. Skin is white and shiny and can be raised and thickened (Fig 1). Anal involvement results in a “figure of eight pattern.” Biopsy allows histologic diagnosis and excludes malignancy. Positive histology, as seen in this case, includes degeneration of the basal cell layer of epidermis, edema, homogenization of upper dermis collagen, and a band-like inflammatory infiltrate in the lower dermis.

First-line conventional management is ultra-potent topical steroid, though there is no consensus about optimal regimen.^5^ Moisturizers soften skin, minimizing cracking and erosions. Oral and intralesional steroids have been used, along with other potent topical immunosuppressants such as tacrolimus.^6^ Concurrent yeast infections may require antifungal treatment. Surgical scar release (adhesiolysis) and excision with healing by secondary intent have been advocated when scarring is symptomatic and medical treatments fail.^5^ Malignancy should be excluded in these cases. Cryotherapy and laser treatment have also been utilized.^7^ However, these techniques can be debilitating, prone to recurrence, and lead to incomplete resolution of symptoms with low satisfaction.^7^

**Figure 2:**
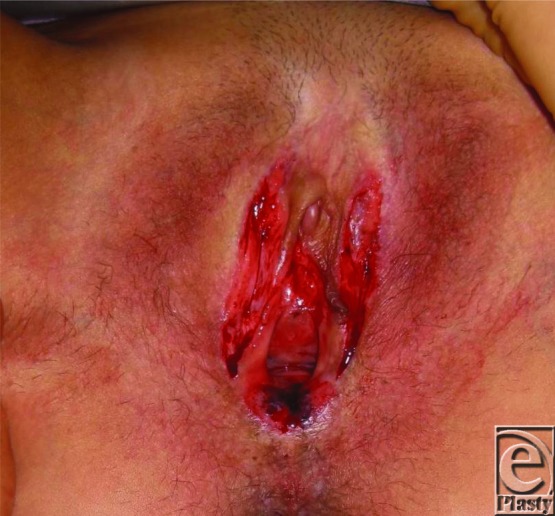
Postoperative excision of vulval lichen sclerosus illustrating release of the stenosis of the introitus.

**Figure 3:**
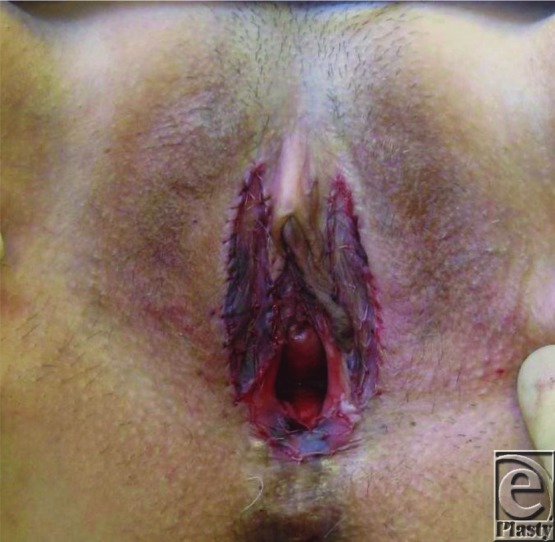
Sheet split skin grafts secured to the areas of excised vulval sclerosus.

**Figure 4:**
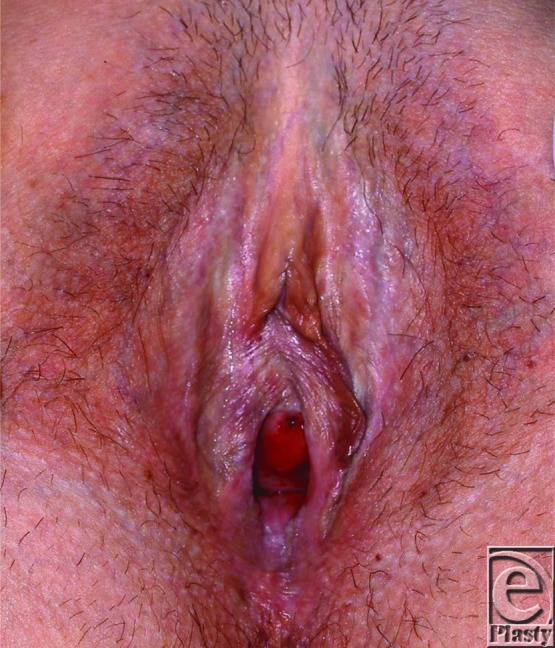
Postoperative images at 6 months showing healed areas of split skin graft and maintenance of patency of the introitus compared with preoperatively.

Vulval LS recalcitrant to medical treatment or that recurs following simple excision or adhesiolysis may successfully be treated with wider excision and reconstruction with SSG.^8^ In our department, 6 such cases referred to a single plastic surgeon were treated with wider excision and SSG (Figs 2-4). Average scores using visual analog scales showed a reduction in pain (from 4/10 to 1/10), a reduction in bleeding and cracking (from 4.3/10 to 0.6/10), and improvements in ability to have sexual intercourse (from 1.5/10 to 6.25/10). Patients also noted fewer episodes of feelings of tightness and improved urinary stream. Average follow-up was 26 months (range, 2-58 months). These findings are in keeping with other studies.^7^^,^^8^

Vulval LS is a chronic, relapsing, debilitating disease. When topical steroid treatment fails, simple excision or adhesiolysis can still lead to disfiguring outcomes, with low satisfaction rates and symptom recurrence.^7^^,^^8^ Involvement of the plastic surgeon in a multidisciplinary setting could select patients who might benefit from wider excision of vulval LS and reconstruction with SSG. This may improve quality of life for these patients, treating their LS more effectively.^8^
